# HAPPE: A Tool for Population Haplotype Analysis and Visualization in Editable Excel Tables

**DOI:** 10.3389/fpls.2022.927407

**Published:** 2022-07-01

**Authors:** Cong Feng, Xingwei Wang, Shishi Wu, Weidong Ning, Bo Song, Jianbin Yan, Shifeng Cheng

**Affiliations:** ^1^Guangdong Laboratory of Lingnan Modern Agriculture, Genome Analysis Laboratory of the Ministry of Agriculture and Rural Affairs, Agricultural Genomics Institute at Shenzhen, Chinese Academy of Agricultural Sciences (CAAS), Shenzhen, China; ^2^State Key Laboratory of Crop Stress Adaptation and Improvement, School of Life Sciences, Henan University, Kaifeng, China; ^3^Shenzhen Research Institute of Henan University, Shenzhen, China; ^4^Hubei Key Laboratory of Agricultural Bioinformatics, College of Informatics, Huazhong Agricultural University, Wuhan, China

**Keywords:** haplotype, SNPs, phylogenetic clustering, visualization, Excel

## Abstract

Haplotype identification, characterization and visualization are important for large-scale analysis and use in population genomics. Many tools have been developed to visualize haplotypes, but it is challenging to display both the pattern of haplotypes and the genotypes for each single SNP in the context of a large amount of genomic data. Here, we describe the tool HAPPE, which uses the agglomerative hierarchical clustering algorithm to characterize and visualize the genotypes and haplotypes in a phylogenetic context. The tool displays the plots by coloring the cells and/or their borders in Excel tables for any given gene and genomic region of interest. HAPPE facilitates informative displays wherein data in plots are easy to read and access. It allows parallel display of several lines of values, such as phylogenetic trees, *P* values of GWAS, the entry of genes or SNPs, and the sequencing depth at each position. These features are informative for the detection of insertion/deletions or copy number variations. Overall, HAPPE provides editable plots consisting of cells in Excel tables, which are user-friendly to non-programmers. This pipeline is coded in Python and is available at https://github.com/fengcong3/HAPPE.

## Introduction

The decrease in DNA sequencing costs and routine bioinformatics platforms have led to the explosion of genomic datasets in recent years. To date, more than 700 plant reference genomes have been sequenced and assembled (Marks et al., 2021; Sun et al., 2022). Furthermore, the size of the plant population used for resequencing studies increases dramatically, resulting in the rapid accumulation of plant genomic datasets. Recently, the genomes of as many as 3,365 accessions of chickpea have been sequenced (Varshney et al., 2021). The reductions in sequencing costs have also promoted populational genomic studies of plants with a large genome, such as gingko (∼ 9.8 Gb) (Zhao et al., 2019) and common wheat (∼16 Gb) (Cheng et al., 2019; Hao et al., 2020; Zhou et al., 2020).

The nearby single nucleotide polymorphism (SNPs) sites in the genome are usually inherited together, and the combination of SNPs is termed the haplotype (The International HapMap Consortium, 2003). The identification of haplotypes is important in the analyses of genomes because it is beneficial for reducing the cost of genotyping, reducing the complexity of association studies, and providing a foundation for haplotype-designed breeding. Haplotype-based association studies have been demonstrated to be efficient in the identification of genes related to complex traits (Todesco et al., 2020). Many tools have been developed to characterize and visualize the haplotypes in genomes. However, the increase in either population size or genome size has caused some problems in displaying genome haplotypes. Although some of the existing tools, such as VIVA (Tollefson et al., 2019), inPHAP (Jäger et al., 2014), and Haploscope (San Lucas et al., 2012), can efficiently produce heatmaps or plots showing the haplotypes in the genome, they rarely display the genotypes with details for each single SNP or any other values or information that can be essential for the researchers to better understand the results. Several interactive tools have been developed to allow zooming in to obtain more details of the haplotypes, but outputting these figures is also not user-friendly.

Here, we present a tool, HAPPE (Haplotype plot in Excel table), which generates visuals of the haplotypes and genotypes in plots consisting of colored cells and/or their borders in Excel tables in a phylogenetic context clustered by a clustering algorithm (aggregative hierarchical clustering). HAPPE automatically detects the haplotypes and displays the genotypes of each single SNP using a distinguished color system in the cells of Excel for any given gene or genomic region of interest. HAPPE also allows the parallel display of other customized information, including the *P* values of genome-wide association studies, the entries of genes or SNPs, and the sequencing depths at each position, thereby enabling the visualization and detection of copy number variations. The users can easily select and copy the values out of the tables to recreate new plots or for the purpose of secondary analyses. Overall, the clustering, characterization, and display of genotypes and haplotypes in editable cells in Excel tables provides an easier method for users to read and access datasets.

## Methods

### Data Input

Several dependences should be preinstalled before the installation of HAPPE: bcftools (Danecek et al., 2021), bgzip and tabix in htslib (Bonfield et al., 2021). HAPPE can take input files of genotypes in a format of VCF compressed with bgzip along with its corresponding index file. A list of samples together with their colors should be provided. HAPPE can also take an input of sequencing depth at the loci to be displayed, but these values need to be calculated in advance using a third-party program, such as a mosdepth (Pedersen and Quinlan, 2018).

### Analysis

HAPPE filters and processes the samples and variants according to the list of samples and genomic regions if they were provided by the users; otherwise, it keeps all samples and variants by default. The annotation information is then extracted from the INFO field for each variant followed by the conversion of genotypes to a numeric matrix with −1, 0, 0.9 and 1 representing reference allele, data not available, heterozygous allele and alternative allele, respectively. The distance matrix between samples is measured by Euclidean distance, and the linkage matrix is then calculated using the ward linkage method. Using this method, a tree is generated, and the samples are grouped using dynamicTreeCut (Langfelder et al., 2008). HAPPE can also calculate the normalized depth of each window (50 bp by default) following the formula:


$$\mathrm{Normalized}\,\mathrm{depth}\,\mathrm{of}\,\mathrm{this}\,\mathrm{window}=\frac{\begin{array}{c} t\,o\,t\,a\,l\,d\,e\,p\,t\,h\,o\,f\,t\,h\,i\,s\,w\,i\,n\,d\,o\,w\\ /w\,i\,n\,d\,o\,w\,s\,i\,z\,e \end{array}}{a\,v\,e\,r\,a\,g\,e\,d\,e\,p\,t\,h\,o\,f\,t\,h\,i\,s\,s\,a\,m\,p\,l\,e}$$

### Visualization

HAPPE uses the openpyxl module^1^ to edit Excel tables. HAPPE first generates a phylogenetic tree in Excel, the width of which can be customized (1,000 columns of Excel cells with a consistent width by default). Next, the label and annotation of samples are displayed, and the cells are correspondingly colored according to the color code provided. Then, the genotypes and the annotation of each variant are displayed and colored blue or red, representing the presence and absence of the variant, respectively. Finally, a heatmap is produced on the right to show the variation in sequencing depth at each window.

### Datasets

We used a published dataset of *Setaria viridis* (Mamidi et al., 2020) to test this pipeline. We first downloaded the reads from the National Center of Biotechnology Information under the accession numbers BioProject PRJNA560514 and PRJNA265547, mapped the reads to the reference genome and called SNPs following the methods described in the manuscript (Mamidi et al., 2020). Finally, two genes (Sevir.5G085400 and Sevir.5G394700) were selected for visualization using HAPPE.

## Results

### Framework

**FIGURE 1:**
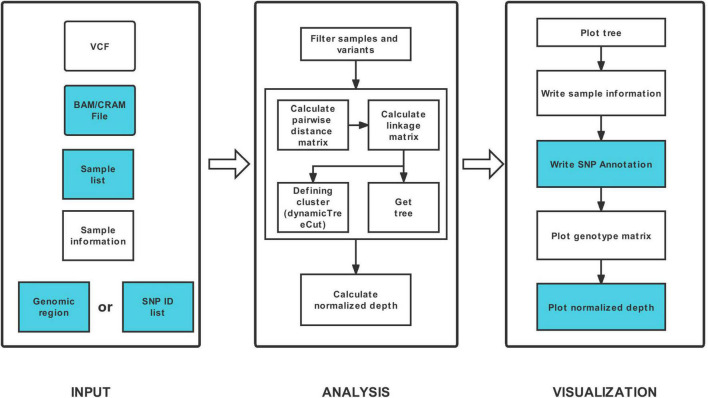
Workflow of HAPPE. The inputs and outputs colored in white are required, whereas the others are optional.

**FIGURE 2:**
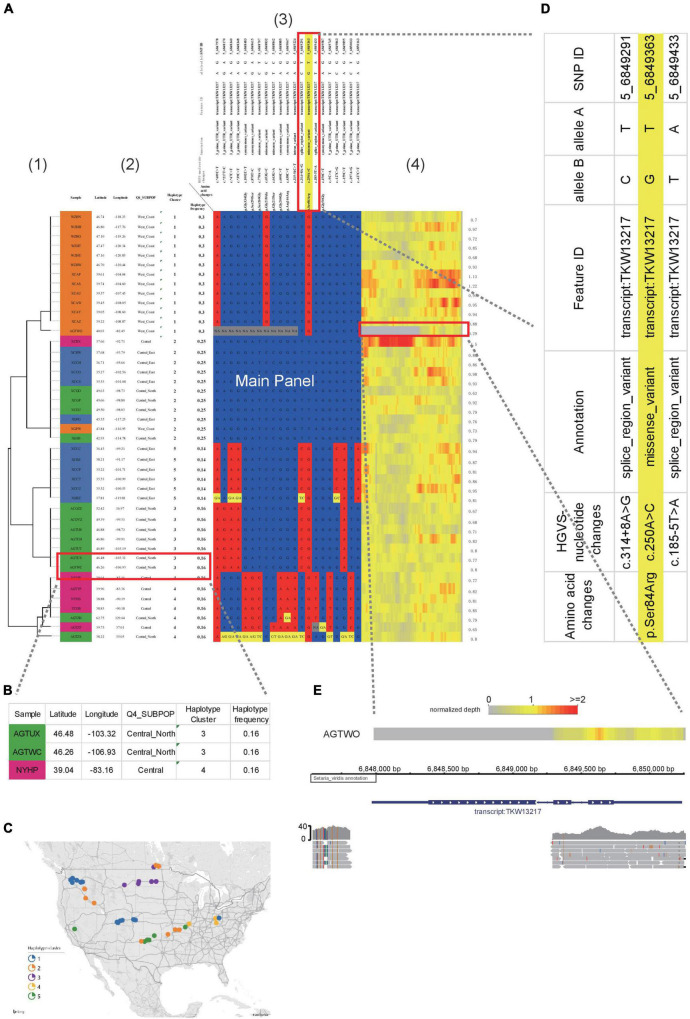
Example of HAPPE on the display of *S. viridis* gene Sevir.5G085400. **(A)** Haplotypes of the *S. viridis* gene Sevir.5G085400, which shows additional information, including (1) a tree, (2) descriptions of samples, (3) annotations of SNPs and (4) a heatmap of read depth. **(B)** Magnified view of Panel 2 showing the descriptions of the samples. **(C)** A map generated using the geographical coordinates shown in the table. **(D)** Zoomed-in view of Panel 3 showing the annotations of SNPs. **(E)** A deletion event shown by the heatmap of read depth in Panel 3, which was further confirmed in a genome browser. More details can be found in Supplementary Tables 1–3.

HAPPE consists of three major steps: data input, analysis and visualization (Figure 1). The tool takes one compulsory input file, the genotyping dataset in compressed Variant Call Format (VCF), and several other optional input files to display additional information of the individuals or SNPs. For example, a file of read alignment in “bam” or “cram” format is required to display the sequencing depth (Figure 2). The genotyping datasets can be either annotated or not. If the VCF file has been annotated by SnpEff (Cingolani et al., 2012), the annotations can be displayed in the plot and tables. Each individual should be given a unique identity, and additional information, including ancestral group, geographical location and read depth, can also be added by setting the corresponding options (see the usage of HAPPE).

The samples and variants are then filtered according to the input list of samples and the range of coordinates followed by the computing of pairwise distance between samples. The distance matrix is then used to calculate the linkage between samples. A linkage matrix is built, and a tree is generated to show the relationship between the samples.

Three compulsory regions, a tree, a list of samples and a main panel of genotype matrix, are displayed in the output table of HAPPE. Additional information of samples, including ancestral group, geographical location and read depth, and annotation of SNPs, including putative effects, gene names, amino acid changes and gene functions, can also be displayed if the corresponding options are properly assigned.

### Examples

To better illustrate the features of HAPPE, we applied it to a subset of the *S. viridis* genomic dataset (Mamidi et al., 2020). Sevir.5G085400, a gene associated with seed shattering, was selected as an example. The genotyping dataset in VCF format was input into HAPPE, which selects the regions according to the coordinates assigned in the options “-k” and “-r.” The samples were then automatically clustered according to the genotypes in the selected regions, and a tree was drawn on the left of the main panel by coloring the cell border on the path of the tree into black. The number of cells used to show the tree is automatically adjusted proportionally to the branch length of the tree.

Other than the main panel showing the haplotypes, the plot can be extended to display additional information by setting a customized option “-i” to add features to the samples and an option of “-I” to add features to SNPs, such as the name and functions of the genes in which the SNPs are located. In the example shown in this study (Figure 2A), we added the labels of individuals in the population and the locations where they were collected shown in latitude and longitude (Figure 2B), allowing a quick projection to the world map in Excel to generate a more direct view of the geographical distribution (Figure 2C) of the individuals with different haplotypes. For the SNPs, we added the substitution type, annotation as well as the gene names and functions (Figure 2D). These factors are critical and allow the readers to have a quick view of the potential effects of the genotypes and haplotypes. A heatmap showing the sequence depth of each SNP in different individuals is shown on the right of the main panel, which can be helpful for the detection of copy number variants or the identification of problematic samples. In the example shown in Figure 2, a deletion event of this gene was detected in the accession AGTWO, as evidenced by the low depth of reads (Figures 2A,E). This deletion was also confirmed in the genome browser Integrative Genomics Viewer^2^.

**FIGURE 3:**
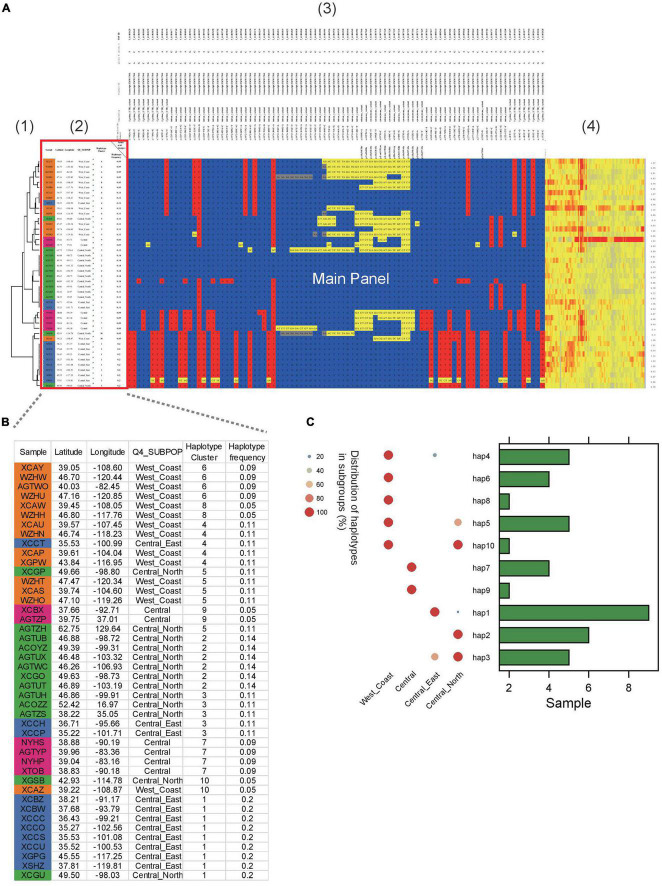
Example of HAPPE on the display of the *S. viridis* gene Sevir.5G394700. **(A)** Haplotypes of the *S. viridis* gene Sevir.5G394700 with additional information, including (1) a tree, (2) descriptions of samples, (3) annotations of SNPs and (4) a heatmap of read depth. **(B)** Zoomed-in view of Panel 2 showing the details of the samples. **(C)** Distribution of haplotypes of the gene in different subgroups. More details can be found in Supplementary Tables 4–7.

In another example, ten haplotypes were identified in Sevir.5G394700, a gene conferring the angle of *S. viridis* leaves (Figure 3). Several of the haplotypes were group specific. For example, haplotype 1 was predominantly found in the “Central_East” subgroup (Figures 3B,C), whereas haplotypes 6 and 8 were specific to the “West_Coast” subgroup. In addition, haplotypes 7 and 9 were specific to the “Central” subgroup.

## Discussion

Genome sequencing and analysis were predominantly performed by bioinformaticians or programmers, but this situation has changed considerably. With the continuous reduction in DNA sequencing prices, whole-genome sequencing is now routinely applied in the works of many clinicians and breeders who are not well-versed in the coding and visualization of datasets. Instead, these researchers favor the use of Excel more than R, Python or any other coding languages for data analysis and visualization. In recent years, more emphasis has been placed on deep collaborations between wet and dry biologists. In these collaborations, the bioinformaticians can help visualize the datasets. However, these visuals are usually generated in an image format, such as png and pdf. Thus, it is difficult for the readers to identify more details, particularly when datasets are large. Although the plots can also be generated into svg format allowing infinite zooming in, the real-time data cannot be shown in the plot either, which potentially hampers the communication and collaboration between different teams. It is occasionally necessary to select a subset of the data for further exploration, but the users have to return to the raw tables to access the data of interest, which could be impossible for large datasets. The tool described in this work reads VCF datasets and outputs the plots consisting of editable cells in Excel tables, enabling the readers to access the data directly from the plot. The additional information can also be displayed in the extension regions (Figures 2, 3) either by setting corresponding options in the commands or editing the table directly in Excel tables, so all the information of samples or the annotation of SNPs can be shown in the plot and table. This feature is very friendly to the non-programmer readers.

### Code Availability and Usage of HAPPE

The application of HAPPE depends on the preinstallation of several tools, including bcftools and bgzip, the path of which can be given in a config file. HAPPE reads genotype datasets in compressed VCF format and selects the samples according to a list provided to the parameter of “-k” and the sites according to the list provided to “-r” or “-s.” The description of samples and the annotation of SNPs can be provided optionally through the parameters “-i” and “-I,” respectively. The users also have the option to exclusively keep the SNPs in the coding or non-coding regions or those resulting in changes in amino acids by selecting a parameter among “-f,” “-n” and “-x.” More details of usage can be found on the page of HAPPE^3^. The code of HAPPE is available on github (see text footnote 3) and Python Package Index (PyPI)^4^ and can be installed using the pip command. It should be noted that the number of samples and sites that can be displayed by HAPPE are constrained by the limitation of the Excel table, which allows only 18,278 columns and 1,048,576 rows at most. Thus, the number of SNPs and samples to be shown should be less than 18,278 and 1,048,576, respectively.

## Conclusion

HAPPE produces haplotype plots consisting of editable cells of Excel tables with customized extension information to help readers understand more details of the data shown in the plot. The output, an Excel table, is user-friendly to non-programmer readers and users and facilitates efficient communication and collaboration between different teams. Given the application of WGS in routine research due to the decreasing sequencing price, we believe that HAPPE will be widely used in various studies and collaborations.

## Data Availability Statement

The original contributions presented in the study are included in the article/Supplementary Material, further inquiries can be directed to the corresponding author/s.

## Author Contributions

SC and JY conceptualized the work and revised the manuscript. CF, XW, and SW coded HAPPE. BS, WN, XW, and SW tested the tool. BS and CF wrote the manuscript. All authors contributed to the article and approved the submitted version.

## Conflict of Interest

The authors declare that the research was conducted in the absence of any commercial or financial relationships that could be construed as a potential conflict of interest.

## Publisher’s Note

All claims expressed in this article are solely those of the authors and do not necessarily represent those of their affiliated organizations, or those of the publisher, the editors and the reviewers. Any product that may be evaluated in this article, or claim that may be made by its manufacturer, is not guaranteed or endorsed by the publisher.

## Footnotes

1
https://foss.heptapod.net/openpyxl/openpyxl

2
https://github.com/igvteam/igv-webapp

3
https://github.com/fengcong3/HAPPE

4
https://pypi.org/project/HAPPE/
